# Magnesium: Nutrition and Homoeostasis

**DOI:** 10.3934/publichealth.2016.2.329

**Published:** 2016-05-23

**Authors:** Jürgen Vormann

**Affiliations:** Institute for Prevention and Nutrition, Ismaning/Munich, Germany

**Keywords:** magnesium, magnesium deficiency, diet, regulation, absorption

## Abstract

The essential mineral magnesium is involved in numerous physiological processes. Recommended dietary intake is often not met and a low magnesium status increases the risk for various diseases. Magnesium status is regulated by several magnesium transport systems either in cellular or paracellular pathways. Numerous drugs either interfere with magnesium absorption in the intestines or the reabsorption from primary urine in the kidney. Low magnesium status has been identified as a significant risk factor for several diseases, including type-2 diabetes, cardiovascular diseases, arrhythmias, as well as general muscular and neurological problems. Therefore, an adequate magnesium supply would be of special benefit to our overall health.

In vertebrates, magnesium is the fourth most abundant cation and is essential for every cell [1],[2]. Magnesium is a co-factor in multiple enzymatic reactions, including those involving energy metabolism and DNA and protein synthesis, and it participates in the regulation of ion channels. Magnesium homoeostasis is therefore fundamental to the existence of life. As a divalent cation, magnesium is capable of cross-linking negatively charged components of the cell membrane and the attachment of magnesium ions results in charge shielding of the (negatively charged) cell surface. This gives rise to a drop in the neuromuscular, muscular and cardiac excitability as well as convulsive seizure-inhibiting action in eclampsia by increasing the extracellular concentration of magnesium. In addition, magnesium inhibits the calcium-induced release and action of transmitters (epinephrine, norepinephrine, acetylcholine, prostaglandins, bradykinin, histamine, and serotonin). By this, magnesium can lead to tranquillization, stress blocking and diminution of neuromuscular, muscular and cardiac excitability [3].

Extracellular magnesium accounts for only about 1% of total body magnesium. The normal serum magnesium concentration has been described to be 0.75 to 1.0 mmol/L [4]. These reference values are still in use in many countries even though a value of 0.85 mmol/L has been suggested as a more appropriate lower reference value [5]. Most of the plasma magnesium, approximately 60% to 65%, is ionised or free magnesium. Of the remaining 35% to 40%, 5% to 10% is complexed to anions such as phosphate, citrate, and sulfate, and 30% is bound to proteins (chiefly albumin). Soft tissue contains about one half of the total body magnesium, approximately 470 mmol. The magnesium content of soft tissues varies between 2.5 and 9 mmol/kg wet tissue weight. In general, the higher the metabolic activity of the cell, the higher the magnesium content. Within the cell, significant amounts of magnesium are in the nucleus, mitochondria, and endoplasmic (or sarcoplasmic) reticulum as well as in the cytosol [6]. Most of the magnesium is bound to proteins and other negatively charged molecules such as nucleoside triphosphates and diphosphates (e.g., ATP and ADP) and nucleic acids (e.g., RNA and DNA) in the cytosol, about 80% of the magnesium is complexed with ATP. Only 1% to 5% of the total intracellular magnesium is free ionised magnesium [7]. The concentration of free magnesium in the cytosol of mammalian cells has been reported to range from 0.2 to 1.0 mmol/L, but values vary with cell type and means of measurement. The free magnesium concentration in the cytosol is maintained relatively constant even when the magnesium concentration in the extracellular fluid is experimentally varied above or below the physiological range [8]. The relative constancy of the free magnesium concentration in the intracellular milieu is attributed to the limited permeability of the plasma membrane to magnesium and to the operation of specific magnesium transport systems that regulate the rates at which magnesium enters or leaves cells. Cellular magnesium transport has been studied intensively and a variety of magnesium transport proteins in different human tissues are operating [9]. In most tissues magnesium influx is dependent on TRPM7 (transient receptor potential melastatin ion channel 7). This channel is ubiquitously expressed whereas TRPM6 is only found along the intestine, the kidney nephron and in lung and testis tissues. Total intracellular magnesium content however is determined by a balance of magnesium influx and magnesium efflux. Whereas magnesium influx systems are quite well characterized, much less is known about the mechanisms how magnesium is transported out of the cell. Magnesium mainly leaves the cell through a Na^+^/magnesium exchange mechanism, this system is dependent on Na^+^,K^+^-ATPase activity [10]. Various other magnesium transport systems have been discovered in recent years, however, it is not known how they quantitatively contribute to intracellular magnesium homeostasis [9].

Magnesium balance in the body is controlled by a dynamic interplay among intestinal absorption, exchange with bone, and renal excretion [2],[11] (Figure 1). On average, about one third of the ingested magnesium is taken up into the blood by paracellular and transcellular pathways. It was shown that after ingesting a single dose, all magnesium was either absorbed or excreted between 25 and 81 hours [12]. In states of magnesium deficiency, intestinal uptake mechanisms for this mineral are upregulated as are the mechanisms for reuptake of ultrafiltrated magnesium in the kidney. In magnesium replete persons, these uptake mechanisms are less effective and can even be further down-regulated in persons, who are loaded with magnesium [1],[2]. In addition to this, acid-base balance influences the overall magnesium status and mild acidosis already leads to significantly increased urinary magnesium excretion [13]. Intestinal magnesium absorption is inversely proportional to the amount ingested. Under normal dietary conditions in healthy individuals, approximately 30% to 50% of ingested magnesium is absorbed [14]. Magnesium is absorbed along the entire intestinal tract, including the large and small bowel, but the sites of maximal magnesium absorption appear to be the distal jejunum and the ileum. The colon absorbs only small amounts of magnesium, which may be important in the context of dietary restriction or compromised magnesium absorption in the small intestine. When dietary intake is restricted, fractional absorption of magnesium may increase up to 80%. Conversely, it may be reduced to 20% on high magnesium diets. There is evidence that intestinal magnesium absorption is through paracellular and transcellular pathways [15]. The majority, about 90%, of normal magnesium absorption occurs passively through the paracellular pathway between the enterocytes involving barrier proteins of the claudin family [2]. The rate of magnesium absorption across the intestinal epithelium is dependent on the transepithelial electrical voltage (which is normally about +5 mV, lumen positive with respect to blood) and the transepithelial concentration gradient. The luminal magnesium concentration may be in the order of 1.0–5.0 mmol/L depending on the dietary magnesium content and the presence of anionic chelators. Only the free magnesium moves through the paracellular pathway so that bound magnesium does not contribute to the transepithelial gradient. Serum free magnesium concentration is 0.5–0.7 mmol/L so that there is normally a concentration gradient from the lumen to the blood side. Various poorly understood hormonal and non-hormonal factors acting through a variety of intracellular signals might influence the passive transport.

**Figure 1.**
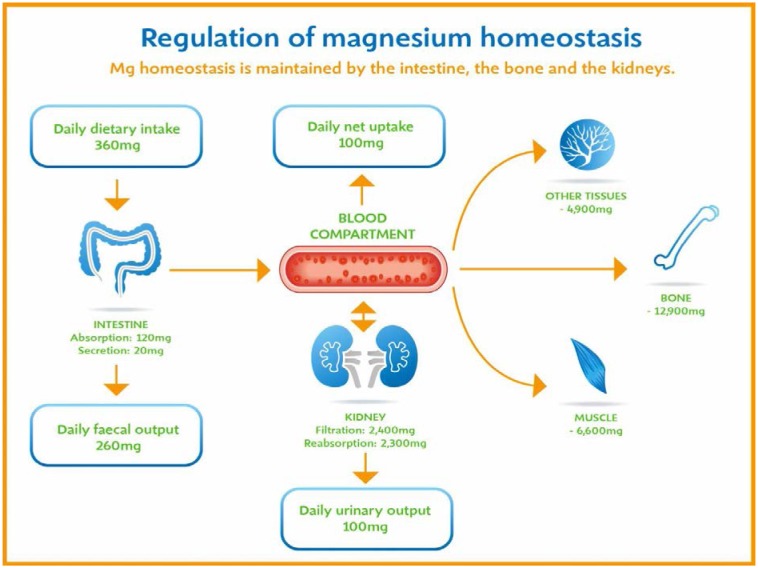
Regulation of magnesium homeostasis.

Other nutrients can affect intestinal magnesium absorption. High levels of dietary fiber from fruits, vegetables, and grains decrease fractional magnesium absorption [16]. However, diets high in vegetables are magnesium-rich, and the high magnesium content of these diets offsets decreased fractional absorption associated with the higher fiber intake. Many foods high in fiber also contain phytate, which may decrease intestinal magnesium absorption because magnesium binds to the phosphate groups on phytic acid. The ability of phosphate to bind magnesium may explain decreases in intestinal magnesium absorption in subjects on high-phosphate diets [17]. Although dietary calcium has been reported to both decrease and increase magnesium absorption, human studies have shown no effect [14]. It is likely that interactions between Ca^2+^ and magnesium occur at high local concentrations achieved by magnesium or Ca^2+^ supplements but not with usual dietary supply.

Aside of regulated intestinal magnesium absorption the kidney is the principal organ involved in magnesium homoeostasis [18]. Under normal conditions, approximately 80% of the total plasma magnesium is filtered through the glomerulus and most of this is reabsorbed (greater than 95%) as the filtrate passes through the nephrons. Therefore, only about 100 mg magnesium is finally excreted each day with the urine, whereas about 2400 mg of magnesium are filtered by the glomeruli into the primary urine. 5% to 15% of the filtered magnesium is reabsorbed in the proximal tubule. The major site of magnesium reabsorption however is the cortical segment of the thick ascending limb of the loop of Henle, which accounts for 65% to 75% of renal magnesium reabsorption. This magnesium transport is passive involving claudin-16, with movement from the tubular lumen to the interstitium driven by the lumen-positive transepithelial voltage. The transepithelial voltage is determined by Na^+^, K^+^, Cl- cotransport and active Na^+^ reabsorption. Therefore, any changes in these transport mechanisms consequently influence magnesium reabsorption. Magnesium reabsorption in cortical thick ascending limb of the loop of Henle is regulated by a variety of hormones that act mainly by increasing the reabsorption rate. These hormonal responses are mediated by changes in both transepithelial voltage and paracellular permeability. It has been shown that 1,25-dihydroxyvitamin D influences the magnesium transport in the cortical thick ascending limb by downregulating claudin-16 expression at the transcriptional level [19]. The amount of reabsorbed magnesium is mediated by the extracellular calcium/magnesium-sensing receptor (CaSR), which is expressed in the basolateral membrane. Activation of CaSR by increased blood concentrations of Ca^2+^ or magnesium leads to inhibition of salt reabsorption and paracellular Ca^2+^ and magnesium transport in the thick ascending limb, thereby increasing divalent cation excretion. Loop diuretics such as furosemide, which act to inhibit the Cl- pump and subsequently block Na^+^ reabsorption, lead to hypomagnesaemia, as these drugs have a large effect on transepithelial voltage. Magnesium reabsorption in the distal convoluted tubule is about 5% to 10% and is of crucial importance for regulating the final amount of excreted magnesium as there is no evidence for magnesium reabsorption beyond this point. Reabsorption there is mediated by an active transcellular transport mechanism. Magnesium enters the cell across the apical membrane through ion channels. Uptake of magnesium is driven by the lumen-negative potential difference in this region of the tubule. Extrusion into the interstitium probably occurs by a Na^+^-dependent exchange mechanism. The pathways involved in magnesium reabsorption in the distal tubule also involve the TRPM6 channel as in intestine. Mutations in TRPM6 were identified in patients with primary hypomagnesaemia with secondary hypocalcaemia [20],[21].

About 99% of total body magnesium is located in bone, muscles and non-muscular soft tissue [5]. Approximately 50–60% of magnesium resides as surface substituents of the hydroxyapatite mineral component of bone [22],[23]. At reduced plasma concentrations, magnesium can rapidly be released from the bone surface and at increased plasma concentrations, magnesium is bound to the surface [24]. Based on investigations with the stable isotopes ^25^Magnesium and ^26^Magnesium in a kinetic model of magnesium metabolism in healthy men, 24% of the human total magnesium exchanges rapidly; of this 79% turns over in 115 h, representing probably the bone surface pool and the remaining part, which may represent serum and easily accessible extracellular space, in less than 9 h [12]. Bone magnesium, therefore, represents a magnesium reservoir that buffers extracellular magnesium concentration. In humans, this magnesium buffering capacity is reduced with increasing age as over a lifetime nearly half of the magnesium content of bone is lost [25].

Magnesium is almost ubiquitous in foods. The primary dietary sources are whole grain cereals, legumes, nuts, and chocolate. Other vegetables, fruits, meats, and fish have intermediate magnesium content, whereas dairy products and beverages have low magnesium content [24]. In the US, the recommended dietary intake of magnesium is 320 mg/day for adult females and 420 mg/day for adult males reflecting the amount that meets the needs of almost all (98%) healthy individuals [26]. Also in other countries, dietary intake recommendations are generally between 300 and 400 mg magnesium per day for adults. In the European Union, the recommended daily allowance (RDA) is 375 mg/day [27]. In Germany, Austria and Switzerland [28], the recommended dietary intakes are 300-400 mg/day for adults.

Several reports indicate that an increasing proportion of the general population does not consume adequate magnesium and consequently develops hypomagnesaemia. This is probably largely due to the refining and processing of food, which is known to considerably reduce the magnesium content [26]. For example, processing of wheat to flour or brown rice to polished rice reduces the magnesium content by approximately 80%.

Dietary data suggest that the average magnesium intake has declined markedly over the last 100 years. Studies in the US showed that 23.5% of the population had a daily dietary magnesium intake of less than 50% of the recommendations [29]. Similar trends to low dietary magnesium intake were found in UK [30] and Germany [31]. The daily magnesium intakes below recommendations, especially in the group of young women, increased from 25% of the total population to about 50%.

Aside of low dietary intake also other causes lead to magnesium deficiency (Table 1).Various studies showed that the percentage of hospitalised patients with hypomagnesaemia is significant. A particularly high incidence of hypomagnesaemia is observed in intensive care units [32].

Excessive excretion of magnesium into the urine is important cause of magnesium depletion. Renal magnesium excretion is proportional to tubular fluid flow as well as to Na^+^ and Ca^2+^ excretion. Therefore, both chronic intravenous fluid therapy with Na^+^-containing fluids and disorders such as primary aldosteronism, in which there is extracellular volume expansion, may result in magnesium depletion. Hypercalcaemia and hypercalciuria have been shown to decrease renal magnesium reabsorption and are probably the cause of the excessive renal magnesium excretion and the hypomagnesaemia observed in many hypercalcaemic states. An osmotic diuresis will result in increased renal magnesium excretion due to excessive urinary volume.

Osmotic diuresis due to glucosuria can thus results in magnesium depletion, and diabetes mellitus is probably the most common clinical disorder associated with magnesium depletion. The degree of magnesium depletion in patients with diabetes mellitus has been related to the amount of glucose excreted into the urine and, hence, with the degree of osmotic diuresis.

An elevated blood alcohol level has been associated with hypermagnesuria, and increased urinary excretion of magnesium is one factor contributing to magnesium depletion in chronic alcoholism. Metabolic acidosis also impairs renal conservation of magnesium [13]. Lastly, a number of rare inherited renal disorders are associated with magnesium wasting because of impaired renal reabsorption of magnesium [2].

**Table 1.** Causes of magnesium deficiency.

Causes	Description
gastrointestinal disorder	prolonged nasogastric suction/vomiting, acute and chronic diarrhea, malabsorption syndromes (e.g., coeliac sprue), extensive bowel resection, intestinal and biliary fistulas, acute haemorrhagic pancreatitis.
renal loss	chronic parenteral fluid therapy, osmotic diuresis (e.g. due to presence of glucose in diabetes mellitus), hypercalcaemia, alcohol, metabolic acidosis (e.g. starvation, diabetic ketoacidosis, and alcoholism).
renal diseases	chronic pyelonephritis, interstitial nephritis and glomerulonephritis, diuretic phase of acute tubular necrosis, postobstructive nephropathy, renal tubular acidosis, postrenal transplantation.
endocrine disorders	Hyperparathyroidism, hyperthyreosis, hyperaldosteronism, syndrome of inappropriate secretion of antidiuretic hormone (SIADH).
drugs	diuretics (e.g. furosemide, hydrochlorothiazide), aminoglycosides, calcineurin inhibitors (cyclosporin A, tacrolimus), amphotericin B, pentamidine, cisplatin, beta-mimetics, catecholamines, anti EGF-receptor antibodies (cetuximab), proton-pump inhibitors (e.g.omeprazole).

In clinical routine, only plasma-magnesium concentrations are determined, there is still no simple, rapid, and accurate laboratory test to determine total body magnesium status in humans [33]. There is a circadian rhythm concerning plasma-magnesium concentration with higher values in the evening and lower in the morning [34]. In addition, stress, physical performance and acidosis might influence plasma-magnesium by magnesium release from the intracellular compartment. By this, artificially high plasma-magnesium concentrations may occur that could hide an existing magnesium deficit.

At first, a low magnesium intake is compensated by activating magnesium from other compartments within the body (above all bones) [35]. If the overall amount of magnesium contained in the body is depleted, this does not directly result in a reduced plasma-magnesium concentration. It is only when the depot of magnesium has been depleted intensively that the concentration of plasma-magnesium decreases. A plasma concentration below the reference value is therefore indicative of a magnesium deficit but a normal plasma-magnesium concentration does not rule out an overall magnesium deficiency [36].

As a normal serum magnesium concentration does not necessarily rule out the prevalence of deficiency, in clinical practice, magnesium deficiency is primarily diagnosed on the basis of symptomatology. Clinical symptoms of magnesium deficiency may vary but very often are connected to increased neuromuscular excitability. Magnesium deficiency symptoms therefore play a decisive role as an indicator for magnesium therapy [37].

## Manifestations of magnesium deficiency

The main biochemical and physiological manifestations of severe magnesium depletion are summarized in Table 2.

A common feature of magnesium depletion is hypokalaemia [38]. During magnesium depletion there is loss of K^+^ from the cells, which is enhanced due to the inability of the kidney to conserve K^+^. Attempts to replete the K^+^ deficit with K^+^ therapy alone is not successful without simultaneous magnesium therapy.

Hypocalcemia is also a common manifestation of moderate to severe magnesium depletion [39]. The hypocalcaemia may be a major contributing factor to the increased neuromuscular excitability often present in magnesium-depleted patients. The pathogenesis of hypocalcaemia is multifactorial. Impaired parathyroid hormone (PTH) secretion appears to be a major factor in hypomagnesaemia-induced hypocalcaemia. Serum PTH concentrations are usually low in these patients, and magnesium administration will immediately stimulate PTH secretion. Patients with hypocalcaemia due to magnesium depletion also exhibit both renal and skeletal resistance to exogenously administered PTH, as manifested by subnormal urinary cyclic AMP and phosphate excretion and a diminished calcaemic response. All these effects are reversed following several days of magnesium therapy. Vitamin D metabolism and action may also be abnormal in hypocalcaemic magnesium-deficient patients. Resistance to vitamin D therapy has been reported in such cases.

**Table 2.** Major manifestations of magnesium deficiency.

Aspects	Manifestations
biochemical	hypokalaemia, excessive renal K^+^ excretion, decreased intracellular K^+^ , hypocalcaemia, impaired parathyroid hormone (PTH) secretion, renal and skeletal resistance to PTH, resistance to vitamin D.
neuromuscular	positive Chvostek's and Trousseau's sign, spontaneous carpal-pedal spasm, seizures, vertigo, ataxia, nystagmus, athetoid and chorioform movements, muscular weakness, tremor, fasciculation and wasting, headache.
psychiatric	depression, psychosis, migraine.
cardiovascular	electrocardiographic abnormalities,prolonged PR- and QT-intervals, U-waves, cardiac dysrhythmias, atrial tachycardia, fibrillations, torsades de pointes.
gastrointestinal	nausea, vomiting.

Neuromuscular hyperexcitability may be the presenting complaint of patients with magnesium deficiency. Tetany and muscle cramps may be present. Generalized seizures (convulsions) may also occur. Other neuromuscular signs may include dizziness, disequilibrium, muscular tremor, wasting, and weakness [38]. Although hypocalcaemia often contributes to the neurological signs, already hypomagnesaemia alone results in neuromuscular hyperexcitability [3].

Magnesium depletion may also result in electrocardiographic abnormalities as well as in cardiac dysrhythmias, which may be manifested by a rapid heart rate (tachycardia), skipped heart beats (premature beats), or a totally irregular cardiac rhythm (fibrillation) [38],[40]. In a study that provided only 101 mg magnesium/2000 kcal a day to volunteers for up to 78 days, one third of the volunteers developed severe cardiac arrhythmias and had to be replenished with magnesium [41].

## Low magnesium intake or low magnesium status and disease risks

During recent years, several large epidemiological studies have been published suggesting a risk reducing effect of a high magnesium intake and/or a low plasma magnesium concentration with regard to various diseases or an increased risk with low intake.

In epidemiological studies, an inverse correlation between magnesium intake and the risk of developing diabetes mellitus (T2DM) was found [42]–[44]. A recent meta-analysis of epidemiological studies with more than 500,000 participants affirmed a diabetes risk reduction by 14% with every 100 mg increase in daily magnesium intake [45].

The ARIC study [46] also reported a significant reduction of sudden cardiac death in the group of participants with high plasma magnesium concentration. Compared to the lowest quartile the participants in the upper quartile of plasma magnesium concentrations had a 55% lower risk. This result is supported by a case control study in a subgroup of women from the Nurses' Health Study. Also in this study the risk for sudden cardiac death was reduced by 77% if the highest quartile of plasma magnesium concentration was compared to the lowest quartile [47].

These results were also confirmed by a study from Germany [48]. The Study of Health in Pomerania investigated a representative sample of the Northeast German population aged 20 to 79. Over a period of 10 years all occurring deaths in the cohort of 3910 persons were recorded. All cause mortality but especially also cardiovascular mortality was significantly increased by 40% in the group with plasma magnesium concentration below 0.73 mmol/L. This low magnesium concentration was found in 25% of the population.

The National Health and Nutrition Examination Survey Epidemiologic Follow-up Study also showed an inverse relationship of serum magnesium and mortality from coronary artery disease (CAD) [49]. Another study, based on a cohort of 12708 participants of the ARIC study, showed that the average thickness of the carotid wall in women increased with each 0.1 mmol/L decline in serum magnesium levels (*P* = 0.006). A further result of the ARIC study was that low serum magnesium and high serum phosphorus and calcium were independently associated with greater risk of incident heart failure [50].

Generally an increased risk of cardiovascular disease was connected to a low magnesium intake or a low serum magnesium concentration as shown in a meta-analysis of 16 studies with data from more than 313000 individuals [51], a result that was also reported in a different meta-analysis [52].

Guasch-Ferré et al. [53] reported an association between low magnesium intake and increased cardiovascular, but also cancer risk in a Mediterranean population leading to an overall increased mortality risk. The study included 7216 men and women aged 55-80 y from the PREDIMED (Prevención con Dieta Mediterránea) study, a randomized clinical trial with a median follow-up of 4.8 y.

In 2012 a meta-analysis from 7 studies with more than 241000 participants reported that dietary magnesium intake was inversely associated with the risk of stroke [54].

In summary, a wealth of information is currently available from studies incorporating data from hundreds of thousands participants associating an increased disease risk and mortality with low magnesium intake and/or status.

## Conclusions

Magnesium is essential to every cell. It is a co-factor in multiple enzymatic reactions, including those involving energy metabolism and DNA and protein synthesis, and it participates in the regulation of ion channels. Magnesium balance in the body is controlled by a dynamic interplay among intestinal absorption, exchange with bone, and renal excretion. Magnesium is almost ubiquitous in foods. However, several reports indicate that an increasing proportion of the general population does not consume adequate magnesium. Reasons for magnesium deficiency aside of hereditary causes include low dietary intake, gastrointestinal disorders, renal loss, renal diseases, endocrine disorders, and drugs.

In epidemiological studies low magnesium levels or intakes are correlated with the development of type-2-diabetes, cardiovascular diseases and the occurrence of muscular and neurological disorders.

If dietary magnesium intake is insufficient magnesium supplementation is advisable. Of the various magnesium containing supplements, those containing organic magnesium compounds (esp. magnesium citrate) have been shown to be superior to anorganic magnesium salts [55].
